# Phenogenetics of cortical granule dynamics during zebrafish oocyte-to-embryo transition

**DOI:** 10.3389/fcell.2025.1514461

**Published:** 2025-01-30

**Authors:** Priscila García-Castro, Isabella Giambó-Falian, Ingrid Carvacho, Ricardo Fuentes

**Affiliations:** ^1^ Laboratorio de Fenómica y Embriogénesis Temprana (LAFET), Departamento de Biología Celular, Facultad de Ciencias Biológicas, Universidad de Concepción, Concepción, Chile; ^2^ Laboratorio de Canales Iónicos y Reproducción (CIR), Departamento de Medicina Translacional, Facultad de Medicina, Universidad Católica del Maule, Talca, Chile

**Keywords:** fertilization, zebrafish, cortical granule dynamics, oocyte maturation, polyspermy

## Abstract

Fertilization is a critical process in sexual reproduction that involves the fusion of a capacitated sperm with a mature oocyte to form a zygote. Polyspermy, the fertilization of an oocyte by multiple sperm, leads to polyploidy and embryo lethality. Mammalian and non-mammalian oocytes have evolved mechanisms to prevent polyspermy, including fast and slow blocks. The fast block comprises membrane depolarization post-sperm fusion, temporarily preventing additional sperm fusion. The slow block, triggered by cortical granule (CG) exocytosis, involves the release of proteins that modify the *zona pellucida* to form a permanent barrier, avoiding the fertilization by additional sperm. The evidence shows that immature oocytes often fail to prevent polyspermy due to ineffective CG exocytosis, attributed to impaired intracellular calcium increases, lower content of this ion, and incomplete CG migration. The study of how genetic variations lead to observable phenotypes (phenogenetics) during the oocyte-to-embryo transition, have identified several maternal-effect genes in zebrafish involved in CG behavior. These genes regulate various stages of CG biology, including biosynthesis, maturation, and exocytosis. Mutations in these genes disrupt these processes, highlighting the maternal genetic control over CG properties. Zebrafish has emerged as a pivotal model for understanding the evolving genetic regulation and molecular mechanisms underlying CG biology, providing valuable insights into fertility and early embryonic development.

## 1 Introduction

Fertilization is an essential process in sexual reproduction in which a capacitated sperm interacts with a matured oocyte/egg to form the zygote [Review in (Clift and Schuh, 2013)]. In most animal species, fertilization must be monospermic, and only one sperm penetrates the oocyte [Reviewed in (Liu, 2011; Rojas et al., 2021)]. Fertilization by more than one sperm or polyspermy causes polyploidy, a condition that is generally lethal to the embryo [Reviewed in (Fahrenkamp, Algarra and Jovine, 2020)]. Human eggs can be fertilized by multiple sperm and around 10% of spontaneous abortions are due to triploidy (Hassold, 1980). To ensure monospermic fertilization, oocytes from most mammals have developed a series of mechanisms during evolution that block polyspermy at the level of plasma membrane or their extracellular coat, called zona pellucida (ZP) in mammals and vitelline envelope in non-mammals. As a mechanism firstly described in invertebrates, polyspermy is prevented by modifying the electrical properties of the plasma membrane (PM), where fusion of the first sperm causes its depolarization, thereby preventing fusion of further sperm (Jaffe et al., 1982). Second, fertilization triggers the exocytosis of cortical granules (CGs), a process termed ‘cortical reaction’ (Szollosi, 1967). In some animals, such as sea urchin, starfish, *Urechis*, and frogs, the initial defense against polyspermy is the fast block, or electrical block, which involves a positive shift in the egg membrane potential upon fertilization (Cross and Elinson, 1980; Gould-Somero, Jaffe and Holland, 1979; Grey, Bastiani, Webb and Schertel, 1982; Jaffe, 1976; Miyazaki and Hirai, 1979). Within 1–3 s after the first sperm binding, the membrane potential shifts from a resting level of −70 mV to +20 mV (Gould and Stephano, 1987; Longo, 1986). This rapid depolarization creates a transient environment that prevents additional sperm from fusing with the egg (Carroll and Epel, 1975; Jaffe, 1976). In these species, it is hypothesized that positive membrane potentials during fertilization inhibit further sperm entry, while negative potentials can lead to polyspermy (Gould and Stephano, 1987). This temporary depolarization provides critical time for the establishment of the slow block to polyspermy, which involves activation of the egg’s signaling pathways to ensure a permanent barrier against sperm entry (Jaffe, 1976; Nuccitelli and Grey, 1984). However, unlike these species, in fish, salamander and mammals the electrical block to polyspermy has not been demonstrated (Charbonneau, Moreau, Picheral, Vilain and Guerrier, 1983; Jaffe, Sharp and Wolf, 1983; Lim, 1978; Nuccitelli, 1980). In mammals, such as hamsters, changes in membrane potential involve recurring hyperpolarization as a result of the opening of calcium (Ca^2+^)-activated potassium channels (Miyazaki and Igusa, 1981). In mouse eggs, the membrane potential remains constant during the 60 min following insemination, suggesting the absence of an electrical block to polyspermy (Jaffe et al., 1982). Yet, it has been shown functional expression of voltage-gated calcium channels (Peres, 1987), and of the two members of the Transient Receptor Potential (TRP) channels, TRPV3 and TRPM7 in mouse oocytes and eggs; however their role in changes in the egg membrane potential and/or CGs dynamics remains to be elucidated (Bernhardt et al., 2018; Carvacho et al., 2016; Carvacho, Lee, Fissore and Clapham, 2013; Lee, Yoon, Lykke-Hartmann, Fissore and Carvacho, 2016; Mehregan, Ardestani, Akizawa, Carvacho and Fissore, 2021). In human eggs, it has been reported the expression of ATP sensitive K^+^ channels (Du et al., 2010). Also, hyperpolarizations, similar to hamster eggs, were shown in response to the injection of human sperm extracts (Homa and Swann, 1994). In sea urchin, after an initial depolarization, the egg membrane repolarizes and then returns to its resting potential, which is essential for the initiation of the slow block to polyspermy (Steinhardt, Lundin and Mazia, 1971; Thompson and Knier, 1983). As the membrane potential is restored, CGs are exocytosed, leading to modifications in the ZP, forming a physical and biochemical barrier that prevents additional sperm from penetrating the egg and maintaining the viability and normal development of the embryo [Reviewed in (Tsaadon, Eliyahu, Shtraizent and Shalgi, 2006)].

**FIGURE 1:**
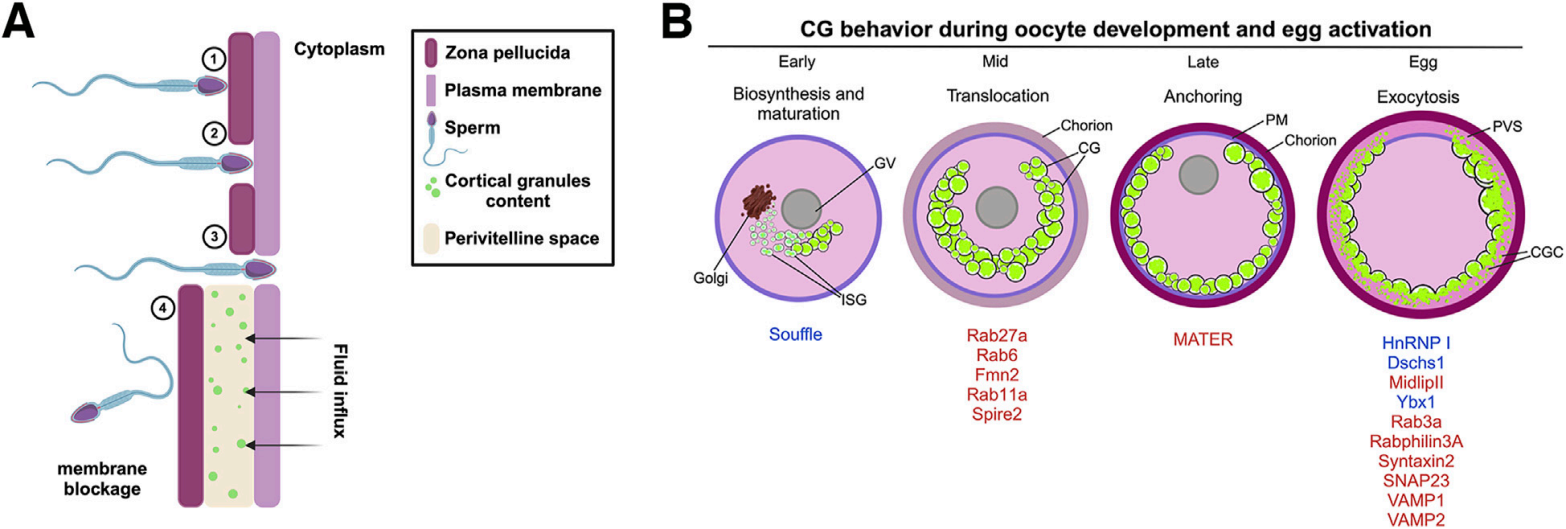
Polyspermy blockage process in mammals and zebrafish maternal proteins involved in cortical granule lifecycle. **(A)** The mouse zona pellucida (ZP) is a glycoprotein matrix composed of several proteins, including ZP1, ZP2, and ZP3, which play key roles during fertilization. 1) Sperm binding and acrosomal reaction: During fertilization, the sperm recognizes and binds to ZP2, triggering the acrosomal reaction. This reaction releases hydrolytic enzymes that help the sperm penetrate the ZP. 2) ZP penetration: As the sperm progresses, it interacts with ZP2, which guides its passage through the extracellular matrix of the ZP. 3) Membrane fusion and nuclear entry: Fusion between the sperm and oocyte membranes enables the sperm pronucleus to enter the oocyte cytoplasm, starting a Ca^2+^ signaling cascade and where it merges with the oocyte pronucleus to form the zygote nucleus. 4) CGE and zona reaction: Following membrane fusion, CGs within the oocyte release their contents into the perivitelline space. This exocytosis induces a zona reaction that reorganizes the structure of the ZP and creates a physical and chemical barrier to block additional sperm from entering, ensuring monospermy. **(B)** CG biosynthesis begins in the early stages of oogenesis, where hypertrophied Golgi units synthesize the vesicles. These vesicles then undergo fusion and maturation, with Suf protein participating in CG maturation. Rab proteins aid in recruiting CGs to the oocyte cortex, assisted by an actin network. The CGs are subsequently anchored to the PM and, under the control of proteins such as HnRNP I, Ybx1, Dschs1 in zebrafish or SNAP23, Rab3a, and Rabphilin3A in mice, exocytose their contents into the PVS, resulting in chorion expansion (or zona pellucida in mammals). ISV, immature secretory vesicle; CGs, cortical granules; PM, plasma membrane; PVS, perivitelline space.

Egg activation, caused by sperm fusion, involves a series of several cellular processes that serves as initial signaling events to initiate the early embryonic development. CG exocytosis (CGE) is triggered by Ca^2+^ oscillations during sperm penetration (Abbott and Ducibella, 2001). This process involves the fusion of CGs with the PM, releasing their contents to the perivitelline space (PVS) (Figure 1A) (Wang, Day and Wu, 2003). CGs are membrane-bound organelles derived from the Golgi complex, which contain diverse proteins including proteases, glycosylated components, cross-linking enzymes, and structural proteins (Liu, 2011; Szollosi, 1967; Wessel et al., 2001). These proteins chemically modify the structure of the glycoproteins that forms and hardens the ZP, creating a barrier to further sperm entry (Figure 1A).

Incomplete oocyte activation refers to the partial or failed initiation of the cellular and molecular processes required for the transition from the oocyte to the embryo upon fertilization. This includes failures in increases of intracellular calcium and the release of CGs. Two hypotheses explain the failure in these events. One suggests that this incomplete activation is related with the meiotic stage of the oocyte. Immature oocytes exhibit a limited number of spontaneous Ca^2+^ transients compared to the mammalian sperm-triggered Ca^2+^ oscillations in mature oocytes. The difference could be potentially explained by a reduced cortical endoplasmic reticulum (ER) area, lower Ca^2+^ storage capacity, and a fewer inositol 1,4,5-trisphosphate (IP_3_) receptors (Wang et al., 2003). The IP_3_ receptor located in the ER, is a critical protein during egg activation, and its activation by IP_3_, generated as product of the hydrolysis of membrane PIP_2_ by the sperm-specific PLCζ, is required for Ca^2+^ oscillations (Miyazaki et al., 1992; Rojas et al., 2021; Wakai et al., 2019). The IP_3_ binds to its receptor on the ER, leading to Ca^2+^ release, CGE and PVS formation (Sharma and Kinsey, 2008). Another hypothesis focuses on the incomplete migration of CGs to the PM during oogenesis, impairing their function during CGE. Additionally, immature oocytes have roughly half the number of functional IP_3_ receptors compared to mature ones (Mehlmann, Mikoshiba and Kline, 1996).

In most mammals, immature and maturing oocytes are more prone to polyspermic penetration during *in vitro* fertilization (IVF), suggesting that these oocytes lack the ability to block polyspermy (Wang et al., 2003). The inability of immature oocytes to release CGs upon sperm penetration is a key factor underlying this susceptibility (Wang et al., 2003). CGs are synthesized in the oocyte center and transported to the PM during meiosis in preparation for fertilization (Cheeseman et al., 2016). Several studies have identified maternal molecular players involved in the transport, docking and exocytosis of CGs. Following egg activation, an increase in cytoplasmic Ca^2+^ triggers the release of CGs into the PVS, modifying the ZP and making it impermeable to other sperm (Carvacho et al., 2018; Liu, 2011; Rojas et al., 2021).

CG biology encompasses biosynthesis, maturation, translocation to the oocyte cortex, anchoring to the PM, and exocytosis upon egg activation. Since oocytes are transcriptionally inactive, these processes rely entirely on the function of maternal gene products (Abrams and Mullins, 2009; Fuentes et al., 2020; Horner and Wolfner, 2008; Lindeman and Pelegri, 2010; Schultz, Stein and Svoboda, 2018). These maternal factors are stored during oogenesis and required for proper embryogenesis and early embryo development [Reviewed in (Conti and Kunitomi, 2024)]. Recent evidence indicates that alterations in maternal-effect gene functions and global translation dynamics can impair CGE and early embryo development [Reviewed in (Lorenzo-Orts and Pauli, 2024; Sun et al., 2018)]. Despite significant advances in understanding the mechanisms of CG biology, polyspermy prevention and embryogenesis, our ability to study these processes comprehensively across different species, particularly in mammals, remains limited. There is a notable scarcity of robust animal models that effectively mimic the full spectrum of these processes.

Here, we focus on maternal genes controlling different aspects of CG biology, from biosynthesis to exocytosis after fertilization or egg activation, including proteins involved in polyspermy blockage, with emphasis on using zebrafish (*Danio rerio*) as an animal model for studying reproductive disorders. Zebrafish oocytes and eggs are easily isolated, manipulated, and contain all the essential maternal factor for correct development, and each step of CG biology occurs at specific oocyte developmental stages. Moreover, zebrafish are well-suited for large-scale genetic and phenotypic screens, providing insights into the role of maternal genes in fertility (Abrams et al., 2020; Dosch et al., 2004; Fuentes et al., 2024; Mullins, Hammerschmidt, Haffter and Nusslein-Volhard, 1994).

### 1.1 Modeling vertebrate fertilization: maternally controlled CG biology in zebrafish

The earliest developmental events in several species, including mammals, sea urchin, fish some insects and plants, are largely controlled by the maternal genome (Flach et al., 1982; Laver et al., 2015). Maternal-effect genes are crucial for oogenesis and embryonic development, influencing processes such as oocyte growth and maturation, pronuclear formation, fusion, the establishment of the first cell division, embryonic genome activation and early embryogenesis (Li et al., 2013; Schultz, 1993). The absence of a single, versatile model system limits our ability to fully unravel the complexities of reproductive barriers, making it challenging to translate findings across species and to understand the cellular and molecular performances of these processes in humans.

In the post-genomic era, zebrafish have emerged as an outstanding model for studying maternal-effect genes due to their genetic similarity to humans and the relative ease of genetic manipulation. Numerous maternal-effect genes have been identified in zebrafish that play crucial roles in oocyte maturation, fertilization, and early embryogenesis (Fuentes et al., 2024; Kanagaraj et al., 2014; Li-Villarreal et al., 2015; Mei et al., 2009; Sun et al., 2018). Although zebrafish and humans have different reproductive strategies -zebrafish possesses external fertilization while humans experience internal fertilization-the underlying cellular and molecular mechanisms are remarkably similar. For instance, both zebrafish and human oocytes undergo CGE to modify the extracellular matrix of the cell and generate a barrier to additional sperm entry and protection (Becker and Hart, 1999; Sathananthan et al., 1985). Zebrafish, with 70% genetic homology to human genes, constitute a pivotal model organism for studying the molecular basis and genetics of numerous diseases [Reviewed in (Briggs, 2002; Fuentes et al., 2018)]. This substantial genetic similarity facilitates the extrapolation of findings from zebrafish to humans, offering profound insights into human biology and disease mechanisms. Consequently, zebrafish have become a prominent model for the investigation of several maternal-effect genes regulating CG biology (Table 1), as will be elaborated in the following sections.

**TABLE 1:** Zebrafish genes involved in cortical granule biology regulation. The table lists the zebrafish and mammalian maternal genes, the corresponding proteins they encode, and the specific processes within CG biology they regulate. The table highlights the genetic factors critical for CG biosynthesis, maturation, translocation, anchoring, and exocytosis during oocyte development and fertilization in zebrafish. Each entry is supported by references to key studies, providing a resource for understanding the molecular underpinnings of CG regulation.

Gene identity	Protein name	Process involved	References
*souffle* ^a^	Suf	CG maturation	Kanagaraj et al. (2014)
*Rab27a*	Rab27a	CG translocation	Cheeseman et al. (2016)
*Rab6a*	Rab6a	CG translocation	Ma et al. (2016)
*Formin2*	Fmn2	CG translocation	Cheeseman et al. (2016)
*Rab11a*	Rab11a	CG translocation	Cheeseman et al. (2016)
Spire2	Spire2	CG translocation	Bernhardt et al. (2011), Jo et al. (2019), Lee et al. (2020)
MATER	MATER	CG docking	Vogt et al. (2019)
*brom bones* (*brb*)^a^	HnRNP I (heterogeneous nuclear ribonucleoprotein I)	CG exocytosis	Mei et al. (2009)
*ybx1* ^a^	Ybx1	CG exocytosis	Sun et al. (2018)
*dchs1b* ^a^	Dchs1	CG exocytosis	Li-Villarreal et al. (2015)
*Rab3a*	Rab3a	CG exocytosis	Masumoto et al. (1996)
*Rabphilin3A*	Rabphilin3A	CG exocytosis	Masumoto et al. (1996)
Syntaxin2	Syntaxin2	CG exocytosis	Iwahashi et al. (2003)
SNAP23	SNAP23	CG exocytosis	Clift et al. (2017), Clift et al. (2018), Mehlmann et al. (2019)
VAMP1	VAMP1	CG exocytosis	de Paola et al. (2021)
VAMP2	VAMP2	CG exocytosis	de Paola et al. (2021)

^a^ Characterized maternal genes in zebrafish.

### 1.2 CG formation: from biosynthesis to maturation

As early oocyte growth progresses, CG are synthesized in the Golgi apparatus and accumulate beneath the oocyte’s PM (Trebichalska et al., 2021). Oogenesis in zebrafish is divided into five stages, distinguished by morphocellular features and oocyte size. CGs are synthesized during stage II and translocated to the oocyte cortex in stage III (Selman et al., 1993). These secretory vesicles are filled with enzymes, carbohydrates and proteins. In sea urchin, for example, CG contain a heterogeneous population of molecules, including hyalin, β-1,3-glucanase, glycosaminoglycans, Ca^2+^, and sulfated acid mucopolysacharides (Bal, 1970; Epel et al., 1969; Hylander and Summers, 1982; Schuel et al., 1974; Wessel, 1989; Wessel et al., 1987). In *Xenopus laevis*, CGs are associated with lectins and Ca^2+^ (Nishihara et al., 1986; Wolf, 1974). In mice, the metalloendoprotease Ovastacin, detected in CGs, is responsible for cleaving a structural glycoprotein ZP2 at its N-terminal portion, which hardens the ZP and prevents polyspermy (Burkart et al., 2012). Ovastacin function is necessary for post-sperm penetration; however, traces of this enzyme seep from unfertilized eggs before fertilization. During this time, its activity is inactivated by fetuin-B, a liver-derived plasma protein [Reviewed in (Stocker et al., 2014)].

CG are initially synthesized as immature secretory vesicles and subsequently mature through fusion with each other (Austin, 1956; Gulyas, 1980). The zebrafish maternal-effect mutant *souffle* (*suf*) was isolated from a forward genetic screen. Eggs from mutant females display defects in chorion elevation and PVS formation, indicative of impaired CG maturation (Kanagaraj et al., 2014). The *suf* gene encodes the zebrafish homolog of SPASTIZIN, which is also mutated in Hereditary Spastic Paraplegia, a neurodegenerative disorder characterized by progressive loss of lower limb motility due to axonopathy of corticospinal upper motor neurons (Blackstone, 2018; Finsterer et al., 2012; Kanagaraj et al., 2014). Post-activation, *suf* eggs retain CGs, leading to the chorion elevation defect. Further analysis revealed that *suf* oocytes possesses and increased number of smaller CGs, with Suf/Spastizin colocalized in a luminal microdomain of these vesicles, indicating its role in their formation (Kanagaraj et al., 2014). This accumulation of immature CGs and a delay in their exocytosis suggest that Suf/Spastizin is essential for proper CG maturation during oogenesis. In addition, *suf* mutant oocytes exhibit an accumulation of VAMP4 and clathrin-coated buds, highlighting Suf/Spastizin’s role in vesicle sorting and maturation. This function is likely mediated through the regulation of Dynamin-dependent fission of clathrin-coated buds, essential for CG maturation (Kanagaraj et al., 2014). Spastizin’s interaction with Beclin-1, a protein required for autophagy, further implicates it in vesicle maturation, with mutations leading to autophagosome accumulation (Sagona et al., 2011; Vantaggiato et al., 2013).

The zebrafish *suf* mutant highlights the importance of proper vesicle formation and maturation during oogenesis (Figure 1B). As CGs mature, they are poised for the next critical phase: translocation to the oocyte cortex. This event, which massively occur in mid oogenesis, is essential for positioning the CGs for eventual exocytosis following fertilization. Understanding the molecular details of CG maturation will provide a complete view into the orchestration of CG functions during early and late oogenesis.

### 1.3 CG translocation mechanisms: insights from Rab proteins and cytoplasmic dynamics

**FIGURE 2:**
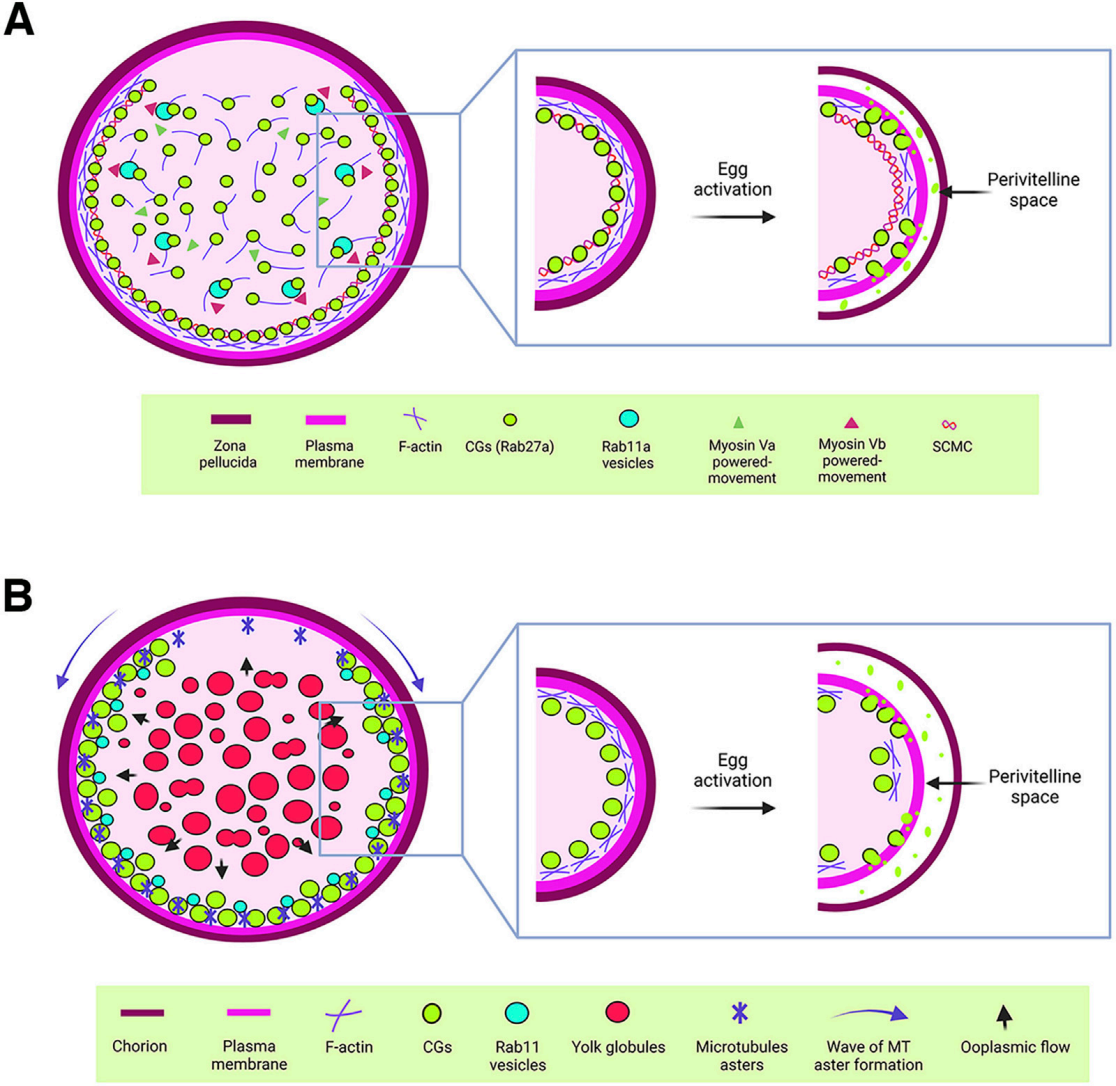
Cytoplasmic components regulating cortical granule biology. **(A)** Schematic representation of a mouse oocyte. In mouse oocytes, Rab27a-associated CGs are synthesized in the central cytoplasm and translocated to the cortex through an actin-mediated mechanism driven by myosin Va. Additionally, Rab11a vesicles facilitate the rapid translocation of CGs to the cortex *via* a similar actin-mediated mechanism, powered by myosin Vb. Once at the cortex, CGs are anchored by the subcortical maternal complex (SCMC). Upon egg activation, cortical actin depolymerizes, enabling the release of CG contents into the perivitelline space. This process modifies the zona pellucida to establish a block against polyspermy. **(B)** Schematic representation of a zebrafish oocyte. CGs are synthesized in the central cytoplasm of the oocyte and translocated to the actin-rich cortex as the oocyte matures. This translocation is driven by a cytoplasmic flow (blue arrows), generated by the fusion of yolk globules. In the cortical region, microtubule (MT) asters, which form in a wave from the animal pole to the vegetal pole during oogenesis, work alongside Rab11 vesicles to ensure the proper exocytosis of CGs. This process is critical for chorion elevation following egg activation. For CGs to release their contents into the perivitelline space, cortical actin must first undergo depolymerization.

CGs are synthesized in the oocyte center and then radially translocated to the cortex to effectively release their content into the PVS after fertilization or activation (Figure 1B). In mice, Rab27a has been identified as a key biomarker of CG translocation (Figure 2A). In fact, transgenic mice of the *Ashen* strain, which lacks functional Rab27a, fail to translocate the CGs to the cortex (Cheeseman et al., 2016). This finding underscores Rab27a’s essential function in CG transport. In mice oocytes, CG trafficking is an actin-dependent but microtubule-independent mechanism powered by myosin Va (Figure 2A) (Cheeseman et al., 2016). Oocytes from *Fmn2* ^−/−^ mice, lacking the actin nucleation factor Fmn2, also display defects in CG translocation; thus confirming the involvement of the actin network in this process (Cheeseman et al., 2016). Rab11a vesicles, responsible for transferrin trafficking in oocytes, have been shown to cooperate in the translocation of CGs to the cortex, with Rab27a vesicles binding to Rab11a to facilitate their movement, dependent of the motor protein myosin Vb (Figure 2A) (Schuh, 2011; Vogt et al., 2019). Another maternal factor, Spire2, has been reported to interact with FMN2 and myosin Va, and it localizes in the oocyte cortex and in Rab11a-positive vesicles. This zinc-finger containing domain Spire2 protein, has a crucial role in oocyte asymmetric division, and actin mesh formation. It has been shown that oocytes treated with Zn^2+^ chelator as TPEN or SpireFull-Zinc^Mut^ injected, exhibited impairment in cortical and cytoplasmic actin network, highlighting the critical role of Zinc homeostasis in actin organization and, later as Zn^2+^ sparks, during egg activation (Bernhardt et al., 2011; Jo et al., 2019; Lee et al., 2020). In sea urchin oocytes, cytoskeleton depolymerization assays also confirmed that actin microfilaments are responsible for CG translocation to the cortex prior to fertilization (Wessel et al., 2001).

In zebrafish, CG translocation is associated with yolk globules (YGs), which fuse and generate an animal pole-directed cytoplasmic flow moving CGs to the cortex as oogenesis progresses (Figure 2B) (Shamipour et al., 2023). Although no direct association has been made between actin filaments and microtubules in this vesicle movement, CGs are associated with Rab11 at the oocyte surface, and this movement depends on peripheral microtubule asters (Figure 2B). In addition, overexpression of a dominant negative variant of Rab11 in zebrafish oocytes leads to defects in chorion elevation and PVS formation, highlighting Rab11’s essential role in CGE (Shamipour et al., 2023).

Recently, the zebrafish maternal-effect mutant *krang* was reported (Fuentes et al., 2024). Eggs from homozygous females fail to elevate the chorion and display delayed CGE after egg activation, retaining CGs in the cortex due to altered translocation dynamics (Fuentes et al., 2024). Also, *krang* transcript localizes with CG in early oogenesis, suggesting a function in their formation and translocation. Remarkably, several maternal-effect genes were also examined *over easy* (*ovy*), *p33bjta*, *poached* (*poac*), and *black caviar* (*blac*). These genes regulate key processes during oocyte maturation and egg activation in zebrafish, specifically are required for early steps in YG sizing, protein cleavage, independent of nuclear maturation (Fuentes et al., 2024). Analysis of CGs in activated mutant eggs revealed significant abnormalities in size, area, and number, which are correlated with defects in CGE and chorion elevation. Specifically, *p33bjta* and *ovy* mutants displayed reduced chorion elevation, with remaining CGs after egg activation, presumably due to defective CG translocation during oogenesis. This finding demonstrates that CG transport and exocytosis are regulated independently, highlighting a complex and parallel coordination of maternal genes functions during oocyte maturation and egg activation.

### 1.4 CGs at the frontier: anchoring with the plasma membrane

To gain exocytosis competence, CGs must anchor to the oocyte PM (Figure 1B). Such a competence is defined as the ability to undergo exocytosis in response to an increase in cytoplasmic Ca^2+^ levels (Ducibella et al., 1993). In mammalian oocytes, a subcortical maternal complex (SCMC) has been identified (Figure 2A), involving four maternal proteins: oocyte expressed protein (also known as FLOPED), transducin-like enhancer of Split 6 (TLE6), K-homology (KH) domain containing 3 (also known as FILIA) and the Nod-like receptor (NLR) family pyrin domain containing 5 (also known as MATER) (Li et al., 2008; Yu et al., 2014). Maternal MATER, in particular, is localized in the oocyte cortex beneath the PM, where it is involved in CG anchoring to the membrane by associating with myosin IIA. Loss of function of MATER in oocytes from null mice leads to defective CG accumulation at the PM, impaired exocytosis, and subsequent polyspermy (Vogt et al., 2019). Cortical actin reorganization is essential for CGs to fuse their membranes with the PM, a process linked to myosin IIA (Figure 2A). After exocytosis, actin also plays a role in compensatory endocytosis to retrieve the CG crypts from the egg surface (Becker and Hart, 1996; Becker and Hart, 1999; Sokac et al., 2003).

Despite our current understanding of the role of MATER and a few other proteins in CG anchoring within mammalian oocytes, our knowledge of additional maternal regulators involved in this process remains limited (Figure 1B). The genetic tractability of the zebrafish offers a unique opportunity to discover new factors involved in GC anchoring, in a different vertebrate system. By leveraging the advantages of zebrafish, we can deepen our understanding of the molecular mechanisms underlying CG anchoring and identify novel maternal factors that may be conserved across species.

### 1.5 The final barrier: CG exocytosis in egg activation and polyspermy prevention

CGs accumulate in the oocyte cortex until fertilization or egg activation, at which point they release their content into the PVS and harden the ZP to prevent polyspermy in mammals or chorion in fish, by fusing their membrane with the egg PM (Figure 1B). Several zebrafish maternal-effect mutants with altered CGE have been described. For example, the *brom bones* (*brb*) mutant eggs present a defect in egg activation, where mutant embryos fail to elevate the chorion due a blocked of CGE. The *brb* gene encodes HnRNP I, an RNA binding protein that regulates cytoplasmic Ca^2+^ levels after egg activation. The mutant phenotype can be rescued by providing Ca^2+^ or IP_3_ to the eggs, indicating the important role of Ca^2+^ signaling in CGE (Mei et al., 2009).

The oocyte cortex is rich in cortical actin, a cytoskeletal network that must reorganize to allow CG release (Becker and Hart, 1996; Li-Villarreal et al., 2015; Mei et al., 2009). Two zebrafish maternal-effect mutants exhibit defects in CGE due to disrupted cytoskeleton dynamics. In the MZdchs1b mutant, embryos show defective chorion elevation due to a delay in CGE (Li-Villarreal et al., 2015). The *dchs1b* gene encodes Dchs1, an evolutionary conserved cadherin (Clark et al., 1995). This mutant displays abnormal actin and microtubules networks. Drug assays in wild-type embryos revealed that incubation with cytochalasin D, an actin depolymerizing drug, phenocopied the delay in CGE, confirming that this process is actin-dependent but microtubule-independent (Li-Villareal et al., 2015). Another maternal mutant, *aura*, shows complete embryo lethality and mid reduction in chorion expansion due to delayed CGE and retention of CGs in the egg cortex. *aura* encodes for Mid1ip11, and this mutant also present alterations cortical actin reorganization (Eno et al., 2016).

The disruptions in Ca^2+^ signaling and cytoskeleton dynamics highlight the importance of properly regulated protein synthesis for successful CG exocytosis. Transcription ceases during oogenesis and resumes only in the early stages of embryo development after zygotic genome activation [Reviewed in (Vastenhouw et al., 2019)]. Therefore, maternal mRNAs are the sole template for protein synthesis, underscoring the need for precise translational control to ensure the correct execution of molecular and cellular events in the time and space for oocyte maturation, fertilization and embryo development (Conti and Kunitomi, 2024; Lorenzo-Orts and Pauli, 2024). The zebrafish *yxb1*mutant also shows delayed CGE (Sun et al., 2018). The *ybx1* gene encodes Ybx1, an RNA-binding protein involved in translational repression, RNA stabilization, and transcriptional regulation (Goodarzi et al., 2015). The absence of Ybx1 in mutant embryos leads to elevated translation, resulting in increased protein production during oogenesis, which may cause defects in CG transport and retarded CGE (Sun et al., 2018).

Rab3A, a GTPase known to control exocytosis in neurons (Schluter et al., 2004), pancreatic and chromaffin cells (Holz et al., 1994; Johannes et al., 1994; Lin et al., 1996; Olszewski et al., 1994; Yaekura et al., 2003), has been proposed as a CG biomarker. It colocalizes with CGs during oocyte maturation in mouse and sea urchin oocytes, and its absence affects CGE (Bello et al., 2016; Conner and Wessel, 1998). Rabphilin-3A, a Rab3A-binding protein with a C2 domain that interacts with Ca^2+^ and phospholipids (Yamaguchi et al., 1993), is also present in the cortical region of mouse oocytes. Due to its localization and interaction with Rab3A, it is proposed that together they regulate Ca^2+^-mediated CGE (Masumoto et al., 1996).

On the other hand, the fusion of secretory vesicles is mediated by SNARE proteins, which are categorized into v-SNAREs (vesicles-associated membrane proteins or VAMPs) and t-SNAREs (Syntaxin and SNAP-25) (Sollner et al., 1993; Weber et al., 1998). These proteins form a trimeric complex known as trans-SNARE, which becomes cis-SNARE after membrane fusion (Jahn and Scheller, 2006). To recycle these proteins for another round of vesicle fusion, the complex must be disassembled by alpha-SNAP and N-ethylmaleimide-sensitive factor (NSF) (de Paola, Bello and Michaut, 2015). Syntaxin2, SNAP23, VAMP1, and Vamp2 are expressed in porcine oocytes (Tsai et al., 2011), and SNAP23 is also expressed in mouse oocytes, where it regulates CGE. Syntaxin4 transcript is expressed in mouse oocytes as well, but its contribution to CGE has not been confirmed (Iwahashi et al., 2003). VAMP1 and VAMP3 are present in the cortical region of mouse oocytes, and both participate in CGE. The action of VAMPs is sensitive to tetanus toxin, which cleaves them and inhibits the fusion of CG membrane to the PM (de Paola et al., 2021).

### 1.6 CG phenogenetics in the zebrafish: the next challenge

In the context of CG biology, phenogenetics involves identifying and studying the genetic mutations that affect CG synthesis, maturation, translocation, anchoring, and exocytosis (Figure 1B; Table 1). By examining these genetic variations and their resulting phenotypic effects, we can elucidate the molecular mechanisms that regulate these critical processes in fertilization and early embryo development. This approach contributes in identifying key genes and pathways that ensure proper CG function and prevent polyspermy, which is essential for successful fertilization and embryo viability.

**FIGURE 3:**
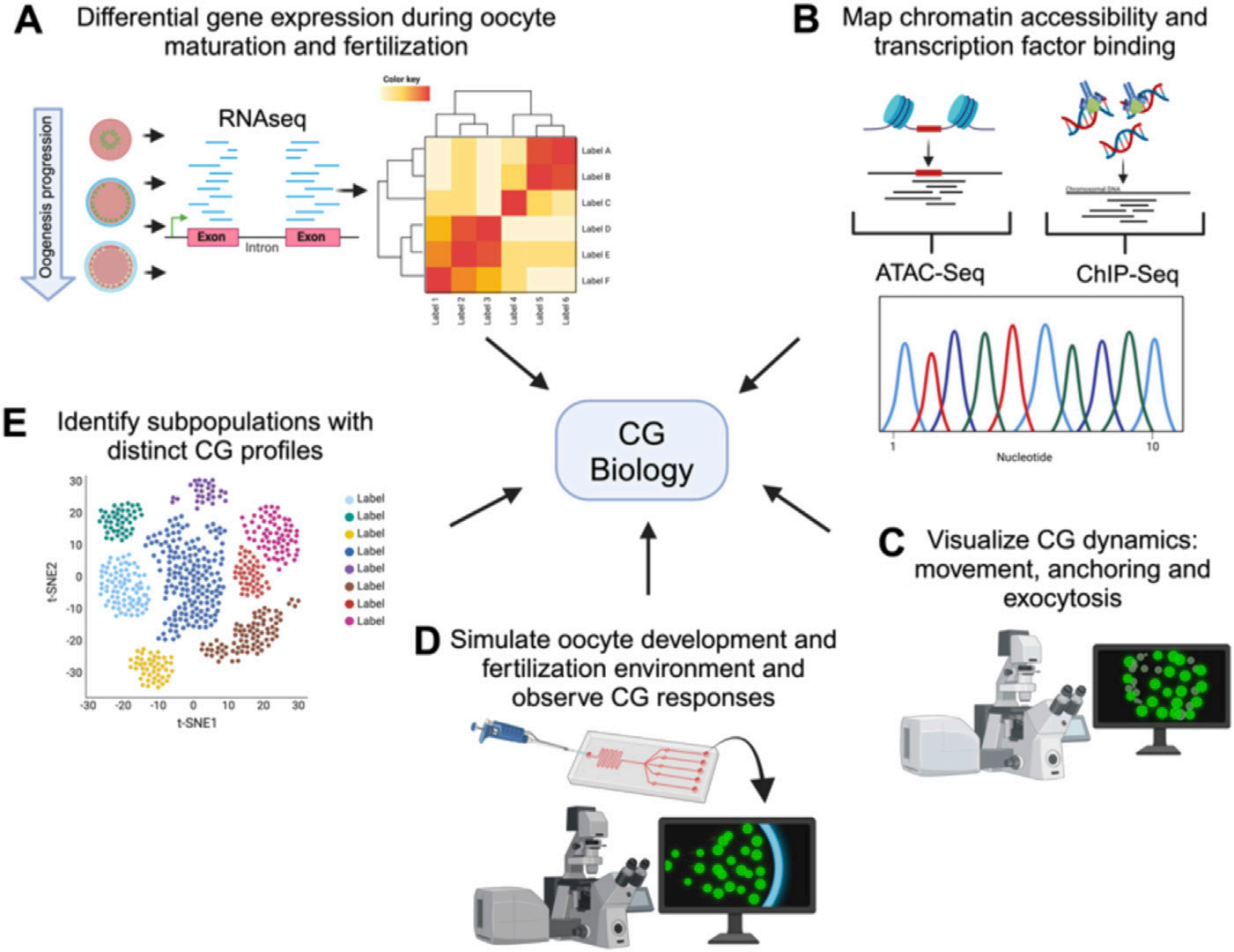
Comprehensive techniques for studying cortical granule biology. Holistic view of the tools available to study the processes involved in CG biology. The figure illustrates various advanced techniques used to examine different aspects of CG biology. Each technique provides unique information into the CG lifecycle, from biosynthesis to exocytosis. **(A)** Transcriptomics analyzes differential gene expression during oocyte maturation and fertilization, offering insights into the molecular regulation of CG biology. **(B)** ChIP-Seq and ATAC-Seq map chromatin accessibility and transcription factor binding sites, elucidating the genetic regulation of CG biology. **(C)** High-resolution live-cell microscopy and Super-resolution imaging allows to visualize CG dynamics, including movement, anchoring, and exocytosis in real-time. **(D)** Microfluidic devices simulate the oogenesis and fertilization environment, allowing observation of CG responses in controlled conditions. Synthetic biology enables the design of constructs to mimic or disrupt CG pathways, facilitating the study of their functional roles. **(E)** Single-cell sequencing identifies subpopulations of cells with distinct CG profiles, highlighting cellular diversity.

Building on the information obtained from phenogenetics, the study of CG biology not only advances our understanding of fertilization and early embryonic development but also has practical implications for assisted reproductive technology (ART) (Cappa et al., 2018). By characterizing the genes, phenotypes and pathways involved in CG regulation, we can address challenges such as the high incidence of polyspermy in IVF and the suboptimal competence of *in vitro* matured mammalian oocytes to undergo CG exocytosis. The aim of studying genes involved in CG regulation is to characterize maternal proteins involved in the vertebrate oocyte-to-embryo transition. On the other hand, integrating advanced techniques will deepen our understanding of CG biology and improve ARTs, ultimately enhancing fertility treatments (Figure 3).

For instance, CRISPR/Cas9 gene editing or TRIM-away can be used to create targeted knockouts and knock-ins in model organisms such as zebrafish and mice to study the role of specific genes in CG dynamics (Clift et al., 2017; Clift et al., 2018; Hang et al., 2016; Rezanujjaman et al., 2024). Recently, two zebrafish knockouts that fail to elevate the chorion have been generated by CRISPR/Cas9. The first is *prss59.1*, a gene upregulated during the induction of ovulation (Klangnurak et al., 2018; Klangnurak and Tokumoto, 2017). The mutation generated a truncated protein of a paralog of trypsin, Prss59.1. Both heterozygous and homozygous embryos display a defect in chorion elevation. Analysis of the chorion by electron microscopy showed that mutant embryos have smaller pores in their chorion compared with wild-type embryos (Rezanujjaman et al., 2024). The other knockout is *val-opsin*, affecting a gene encoding for VAL-opsin (Hang et al., 2016). The progeny of female knockouts also has a defect in chorion elevation upon activation, a high mortality rate, and a delay in hatching (Hang et al., 2016). Using TRIM-Away technology, it was demonstrated that SNAP23 is critical for CGE in mouse eggs (Mehlmann et al., 2019).

Zebrafish maternal null mutants for *larp6a* and *larp6b* were generated using CRISPR/Cas9-and TALEN-based genome editing, respectively (Hau et al., 2020). The La-related proteins (Larps) are a family of evolutionarily conserved RNA binding proteins (Maraia et al., 2017). Eggs from *larp6a* and *larp6a;larp6b* double mutant females display a defect in chorion elevation upon egg activation (Hau et al., 2020). Electron microscopy analysis of oocytes and eggs from double mutant females revealed no alterations in CGs; instead, defects were found in chorion formation and composition (Hau et al., 2020). While it is well-established that failure in PVS formation and chorion elevation post-fertilization or activation is a direct consequence of delayed or blocked exocytosis, CGs were not specifically evaluated in these mutants. Further studies are needed to elucidate if and how these genes are involved in CG biology and their broader role in fertility.

Looking ahead, integrating advanced technologies will significantly enhance our understanding of CG biology (Figure 3). Transcriptomics, including RNA-Seq, can reveal genes identities and differential expression patterns during oogenesis and after fertilization, helping identify key regulatory maternal genes of CG behavior (Bogoch et al., 2022; Fuentes et al., 2024; Smith et al., 2022). Single-cell RNA sequencing can uncover the heterogeneity in gene expression among individual oocytes and eggs, identifying subpopulations with distinct CG-related profiles (Hu et al., 2022; Liu et al., 2022). While CG function is largely regulated by maternal factors stored in the oocyte, the transcriptional and epigenetic landscape established during oogenesis plays a pivotal role in determining the availability and regulation of these genetic factors. ChIP-Seq and ATAC-Seq will provide complementary insights into CG biology by identifying the chromatin accessibility and transcription factor binding events that shape the maternal transcriptome involved in CG biogenesis, translocation, and exocytosis. Although transcription ceases upon initiation of oocyte maturation, the chromatin landscape established earlier determines the maternal mRNA and protein repertoire that supports CG biogenesis, translocation, and exocytosis. Thus, these approaches also have the potential to uncover novel regulatory pathways and maternal-effect genes critical for oogenesis progression and egg activation (Bogoch et al., 2022; Hanna et al., 2022; Liu et al., 2022; Yano et al., 2022; Zhang et al., 2020).

High-resolution live-cell microscopy and super-resolution imaging will provide detailed visualization of CG dynamics, including their movement, anchoring, and exocytosis (Fuentes et al., 2024; Morawiec et al., 2024). On the other hand, classical electronic microscopy of eggs from mutants allows the characterization of pattern, size and morphology of CGs giving insights of gene function. Microfluidic devices can recreate the fertilization environment *in vitro*, allowing for precise control and observation of CG behavior in response to different stimuli (Alias et al., 2021; Wu et al., 2024; Yanez and Camarillo, 2017). Finally, developing engineered living systems could be employed to design and test synthetic constructs that mimic or disrupt CG-related pathways, providing insights into their regulatory mechanisms (Cui et al., 2022; Hilburger et al., 2019). These innovative approaches will open new avenues for research, improving ARTs and broadening our knowledge of reproductive biology (Figure 2).

## 2 Discussion

The mechanisms underlying CG biology are essential for ensuring monospermic fertilization and subsequent embryo development. This review highlights the complex and multifaceted processes involved in CG biosynthesis, translocation, anchoring, and exocytosis, revealing the intricate molecular factors that regulate these stages during oogenesis and fertilization (Figure 1).

To understand polyspermy and fertility disorders, it is essential to uncover the molecular mechanisms driving CG biogenesis, transport, and exocytosis during oogenesis and egg activation. Zebrafish serves as an excellent model for studying CG biology and human reproduction due to their suitability for genetic and phenotypic screens, ease of oocyte isolation and manipulation, and well-defined stages of CG biogenesis and exocytosis (Fuentes et al., 2018; Fuentes et al., 2020). Maternal-effect mutants in zebrafish have revealed critical roles for maternally-loaded factors in CG maturation and function during egg activation ((Fuentes et al., 2020; Rojas et al., 2021). For example, mutations in the *brb*/*hnRNP I* gene impair CGE and chorion expansion by disrupting IP_3_-dependent Ca^2^⁺ release (Mei et al., 2009). Additionally, actin-related proteins such as Dachsous1b, Aura/Mid1ip1l, and Suf influence CG formation and fusogenic activity (Eno et al., 2016; Kanagaraj et al., 2014; Li-Villarreal et al., 2015). Mutants in *ybx1* also highlight the role of translational control in regulating CG biology and egg activation (Sun et al., 2018). These findings establish zebrafish oocyte and egg as powerful tools for identifying key regulators of female reproduction and exploring the genetic basis of CG biology.

The proper translocation of CGs to the oocyte cortex is crucial for their function during fertilization. In mice, Rab27a has been identified as a key regulator of this process. This translocation is actin-dependent and microtubule-independent, driven by myosin Va. Additionally, Rab11a vesicles facilitate CG translocation by recruiting actin nucleation factors and interacting with Rab27a vesicles, enhancing their movement to the oocyte cortex. In zebrafish, CG translocation involves cytoplasmic flow generated by YG fusion, with Rab11-associated vesicles playing a role in CG exocytosis. The *krang* mutant phenotype, which exhibits defective CG translocation dynamics during oogenesis, further emphasize the maternal genetic regulation of this process (Fuentes et al., 2024). However, further studies are needed to fully elucidate the role of the maternal factor Krang in CG translocation. These findings highlight the conserved yet distinct mechanisms of CG translocation across animal species, emphasizing the need for continued research to uncover the full spectrum of regulatory factors involved.

Anchoring CGs to the plasma membrane is essential for their exocytosis competence, a critical step in successful fertilization. In mammals, the SCMC plays a pivotal role in this process. For instance, MATER´s subcellular location at the oocyte cortex and association with myosin IIA, highlights its crucial function for CG docking and subsequent exocytosis. In MATER-null mice, CGs are incorrectly distributed, leading impaired exocytosis. This disruption has significant consequences, including the failure to release key proteins like Ovastacin, which is essential for cleaving ZP2 after fertilization. Despite these insights, our knowledge of the molecular mechanisms underlying CG anchoring is limited, enhancing a pressing need to identify additional regulators involved in this aspect of oocyte development. CG exocytosis, triggered by increases in the intracellular Ca^2+^ following fertilization, is a critical step in preventing polyspermy. Additionally, proteins such as Rab3A and Rabphilin-3A function during CG exocytosis, facilitating Ca^2+^-mediated exocytosis through their localization in the egg’s cortical region and interaction with Ca^2+^ and phospholipids. These mutant phenotypes contribute to dissect the complexity of CG exocytosis regulation and point to the need for further exploration to uncover novel molecular players and pathways that orchestrate this process prior to fertilization.

In mice, inactivation of MGAT1 gene results in oocytes with a thinner ZP compared to wild type, lacking complex glycans in its structure. *Mgat1* encodes for *N*-acetylglucosaminyltranserase I (GlcNAc-TI), which is responsible for the formation of hybrid and complex glycans (Shi and Elliott, 2004). Whether the defect in ZP formation in MGAT1-null oocytes is related to a defect in CG biology remains unexplored and warrants further investigation. Also, functional analysis shows that the IP_3_R is N-glycosylated at its C-terminal region, which suggests a role of N-glycosylation in oogenesis and egg activation (Bosanac et al., 2004; Shi and Elliott, 2004).

The diversity of genes involved in CG dynamics control, raises intriguing questions about the evolutionary conservation and diversification of CG regulatory mechanisms across species, which have been partially addressed through phylogenetic studies examining the fate of the maternal factors. Recently, a phylogenetic survey determined the distribution of maternal regulatory factors across diverse taxonomic groups, using genome data from vertebrates and beyond (Dunn and Ryan, 2015; Rojas et al., 2021). Some regulatory factors, such as Rab and SNARE complex proteins, and HnRNAP I, are well conserved across species (Rojas et al., 2021). However, certain molecular regulators of CGE, such as Ovastacin, MATER, Feutin-b and Ybx1, show restricted distribution. Further comparative studies will be necessary to comprehend the evolution and functional diversification of maternal factors and the genetic control of CG biology (Rojas et al., 2021).

In conclusion, the processes governing CG biology are essential for successful fertilization and embryo development, with integrated roles played by several molecular factors. This review highlights significant advances in CG research, including studies using the zebrafish model, which have illuminated key genes and mechanisms of CG biology (Table 1). Despite these insights, gaps remain, particularly regarding CG biosynthesis, transport and anchoring. The diverse maternal-effect mutant phenotypes examined, including those in zebrafish, underscore the complexity and species-specific nature of CG regulation and reproductive biology. The power of phenogenetics has proven invaluable in dissecting these processes, highlighting the need for continued research to elucidate the full range of molecular actors and regulatory pathways, bridging the gaps in our understanding of CG biology, reproductive mechanisms and their evolutionary implications.
